# A Giant Non-Traumatic Splenic Pseudocyst Successfully Treated With Cyst Aspiration and Partial Cystectomy: A Case Report and Review of Literature

**DOI:** 10.7759/cureus.61110

**Published:** 2024-05-26

**Authors:** Mosa R Abu Sabha, Mariam Thalji, Karim Abu Laila, Mohamad Alhashlamoun, Amer Abu Rumaila, Sami Bannoura

**Affiliations:** 1 Faculty of Medicine, Al Quds University, Jerusalem, PSE; 2 General Surgery Department, Al-Ahli Hospital, Hebron, PSE; 3 Pathology Department, Al-Ahli Hospital, Hebron, PSE

**Keywords:** spleen-preserving surgery, splenectomy, cystectomy, splenic cyst, splenic pseudocyst

## Abstract

Splenic cysts are extremely rare entities that typically result from prior abdominal trauma, infections, and degenerative diseases. They are divided into two categories: true cysts with epithelial lining, and false pseudocysts without epithelial lining, which is more common than true cysts.

We describe here a case of a non-traumatic splenic pseudocyst in a healthy 29-year-old male patient, who presented with left upper quadrant abdominal pain. Physical examination revealed scaphoid abdomen and left hypochondrium fullness. The spleen was uniformly enlarged, smooth, and firm, with mild tenderness. Laboratory testing was normal. An abdominal CT scan showed a huge unilocular non-enhancing cyst occupying the upper part of the spleen, measuring around 16 × 18.5 × 20 cm. The patient was managed with cyst aspiration and partial cystectomy. The histopathological examination findings are consistent with splenic pseudocyst. A one-year follow-up period revealed no complications or recurrence.

Spleen cysts are rare in clinical practice, posing challenges in diagnosis and treatment. Surgical options include partial or total splenectomy, cyst aspiration, percutaneous drainage, partial cystectomy, and marsupialization. The choice depends on the cyst's size, splenic coverage, and relation to the hilum. Recently, spleen-preserving approaches have been favored to avoid life-threatening sepsis.

Non-traumatic splenic pseudocysts present significant diagnostic dilemmas, requiring histopathological examination for definitive diagnosis. Spleen-preserving management is highly recommended to reduce the risk of life-threatening sepsis.

## Introduction

Splenic cysts are extremely infrequent, affecting around 0.07% of the world's population [1,2]. Depending on whether an epithelial lining is present or not, as well as their likely cause, splenic cysts are classified as either true (primary) or false (secondary/pseudocyst). Pseudocyst of the spleen, which is one of the rarest types, is most commonly caused by abdominal trauma; however, it can also be caused by infections such as tuberculosis, malaria, or infectious mononucleosis.

While large cysts are more likely to be symptomatic, smaller cysts are usually asymptomatic and discovered incidentally [1,2]. The symptoms are caused by the progressive compression of the adjacent structures. The gold standard of treatment is splenectomy. Even so, partial splenectomy, percutaneous aspiration, and cystectomy are also described [3].

We present a case of a 29-year-old male, who was found to have a giant splenic pseudocyst with no prior history of abdominal trauma. The patient underwent successful cyst aspiration and partial cystectomy.

## Case presentation

A 29-year-old male patient presented to our hospital with left upper quadrant abdominal pain for one week. The pain was characterized as continuous and dull, increasing with deep inspiration and movement of the trunk particularly to the left. It was associated with drenching night sweating and significant fatigue. The patient reported the same pain one month ago and denied a history of abdominal trauma. The review of systems was unremarkable. He had no history of alcohol or tobacco use and had a free medical and surgical record. The patient's occupation was identified as a painter and he was living in an urban area without pets.

**Figure 1:**
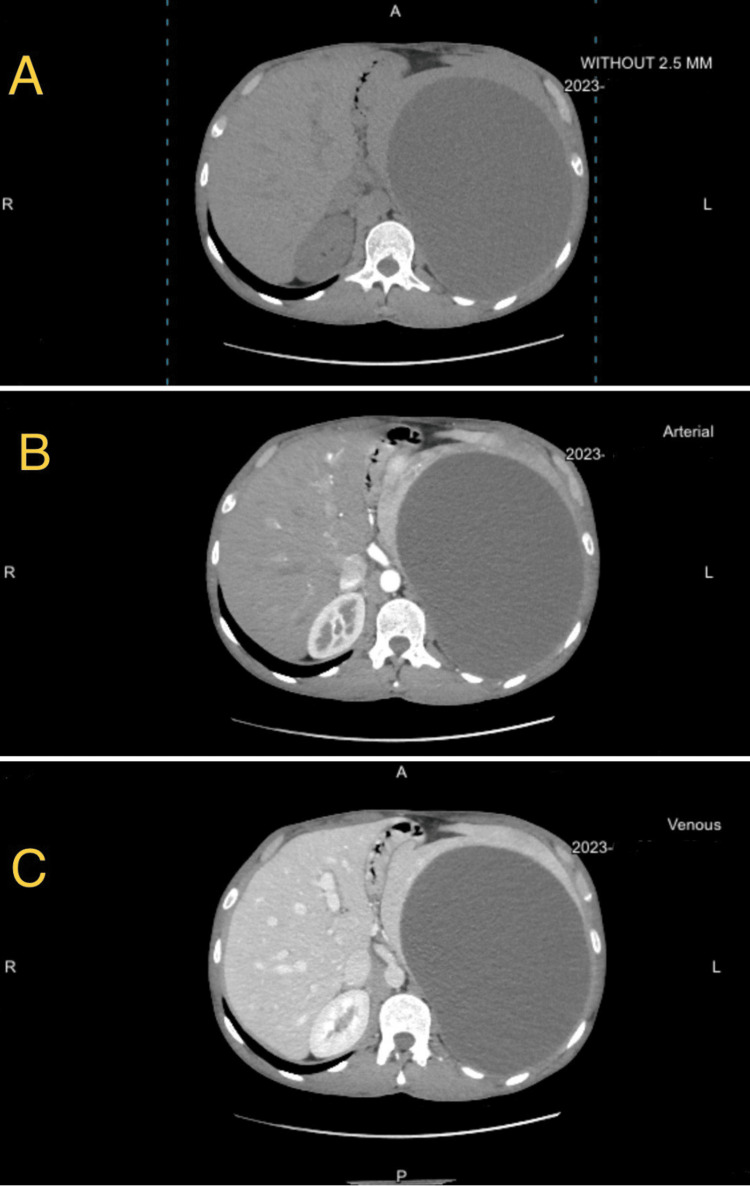
Abdominal CT scan. A. Pre-contrast. B. Arterial phase. C. Venous phase. CT, computed tomography

On physical assessment, the patient was vitally stable. Abdominal examination disclosed scaphoid abdomen and left hypochondrium fullness. On palpation, the spleen was uniformly enlarged, smooth, and firm, with mild tenderness. An abdominal triphasic CT scan with intravenous contrast revealed a huge unilocular non-enhancing cyst occupying the upper part of the spleen, measuring around 16 × 18.5 × 20 cm. This cyst displayed a significant mass effect in the form of marked compression of the stomach and adjacent organs with inferior displacement of the left kidney and upward elevation of the left hemi diaphragm. No calcifications, daughter cysts, or internal membranes were noticed. There was a small (6mm) hemangioma in the left liver lobe with other small liver hemangiomas (Figure 1). The initial laboratory tests were normal Table 1.

**Table 1:** Laboratory tests on admission. Hb, hemoglobin; HCT, hematocrit; RBC, red blood cell; MCV, mean corpuscular volume; WBC, white blood cell; PLT, platelet

Laboratory parameters	Result	Reference range
Hb (g/dL)	15.73	13.5-17.5
HCT (%)	48	41-53
RBCs (million/mm^3^)	5.5	4.3-5.9
MCV (µm^3^)	87	80-100
WBCs (×10^3^)/L)	4.9	4.5-11
PLTs (×10^3^)/µL)	196	150-400

**Figure 2:**
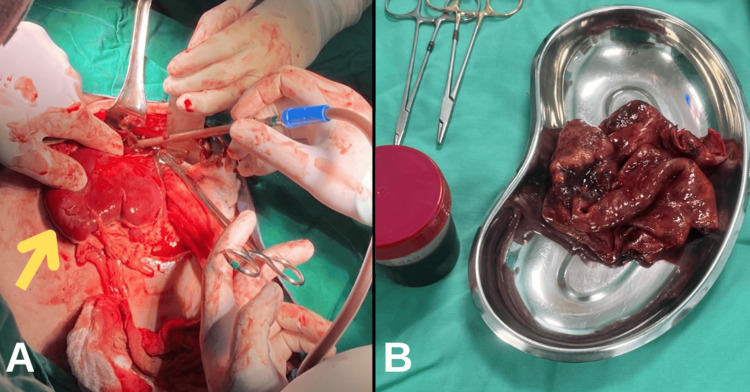
A. Intraoperative image shows the spleen outside the abdomen (arrow). The cyst in the upper pole was dissected and turbid fluid was aspirated. B. The dissected cyst wall.

**Figure 3:**
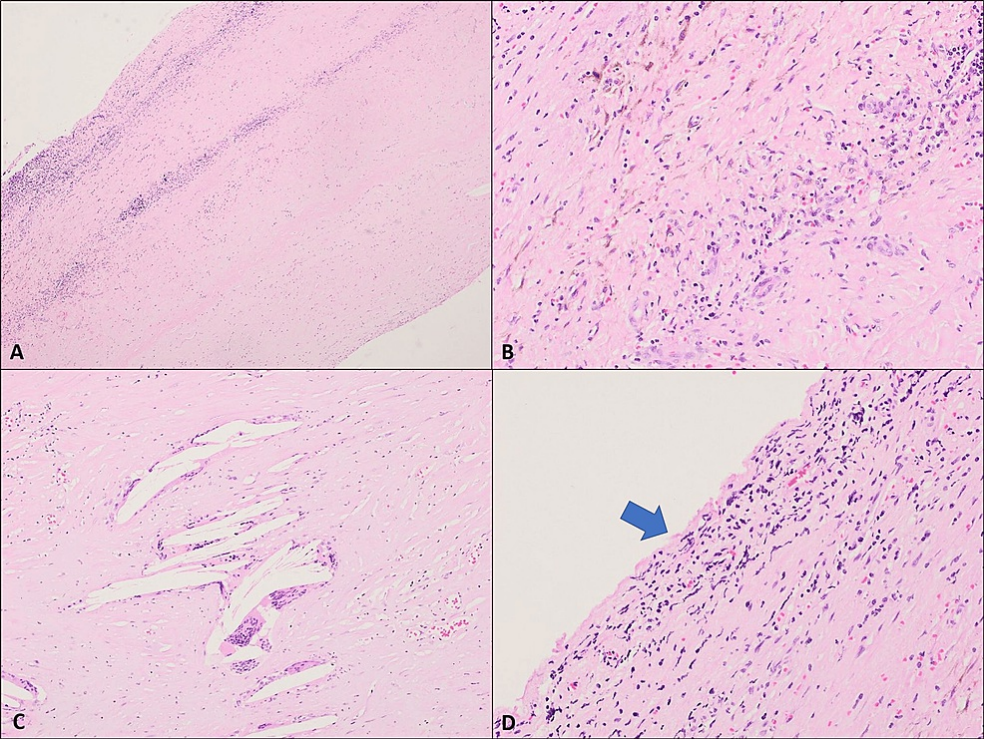
Findings consistent with splenic pseudocyst. A. Sections show a fibrous cyst wall (H&E, 4X). B. Foci of mild chronic inflammation and hemosiderin deposition are seen (H&E; 20X). C. Cholesterol clefts with giant cell reaction are noted (H&E, 10X). D. No epithelial lining is identified (H&E, 20X).

After discussion, the decision was to perform laparotomy and splenic cystectomy with the aim of spleen preservation. The patient was placed in a supine position and under general anesthesia. An upper midline laparotomy was opened layer by layer. A giant splenic cyst was identified and dissected with the aspiration of 4 liters of turbid fluid that was sent for culture. Then, partial cystectomy was performed with preservation of the spleen parenchyma, and two high-vacuum drainages were placed in the cystic cavity and left upper quadrant (Figure 2). The histopathological examination revealed a fibrous cyst wall measuring around 27 × 15 cm with a thickness of 2 mm, with mild chronic inflammation, cholesterol clefts, and foreign body-type giant cell reaction. The specimen was negative for cystic echinococcosis and malignancy. Aspirated fluid was sterile in microbiological culture. These findings were consistent with splenic pseudocyst (Figure 3).

The postoperative period was uneventful, and the patient was discharged home on postoperative day 4 in good general condition. He returned for serial follow-up examinations and ultrasonography. At his 12-month follow-up, he had a good condition without any recurrence or complaints.

## Discussion

We describe a case of a 29-year-old male patient who presented with left upper quadrant abdominal pain, associated with night sweating and fatigue. Physical examination revealed an enlarged, smooth, and firm spleen with tenderness. A CT scan showed a large unilocular non-enhancing cyst causing a significant mass effect. Laparotomy and cyst aspiration with partial cystectomy were performed along with spleen preservation. Histopathological examination confirmed a fibrous cyst wall with mild chronic inflammation, which is consistent with splenic pseudocyst.

**Figure 4:**
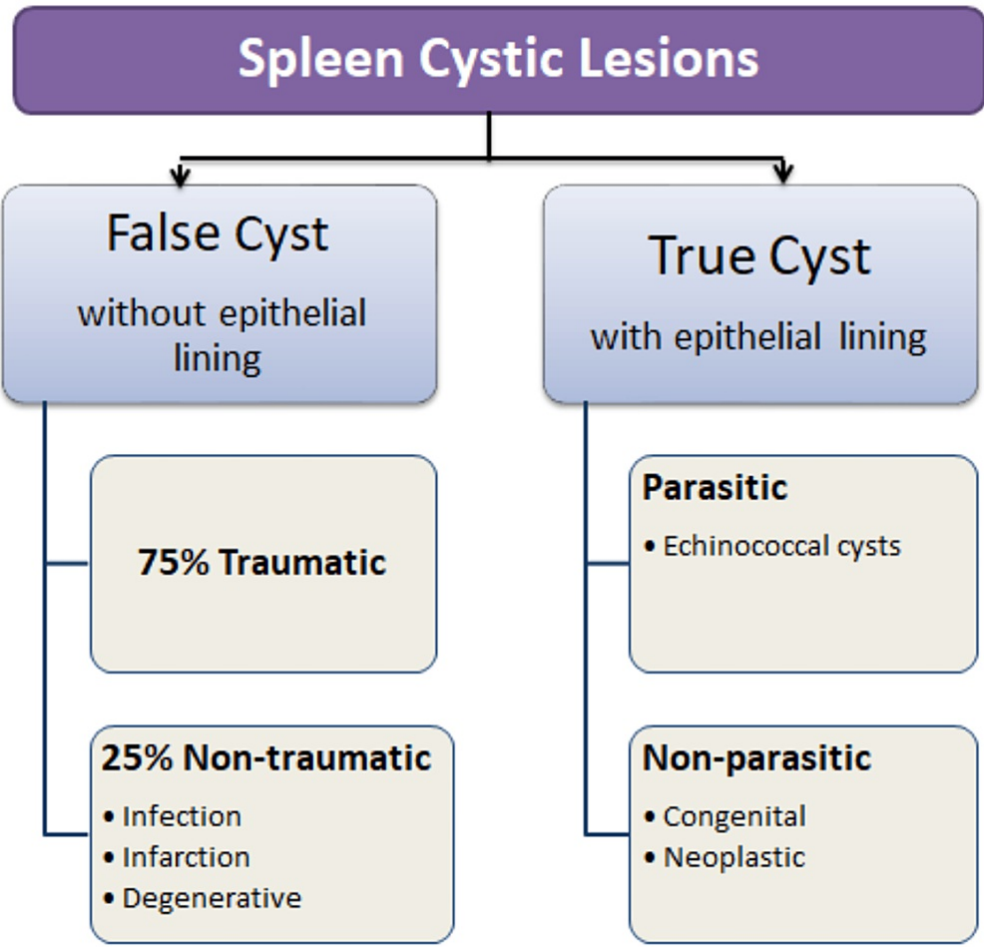
Splenic cysts classification.

Spleen cysts are rarely encountered in clinical practice [3]. Therefore, they present challenges for the surgeons in the work-up and the treatment [4]. Splenic cysts are further divided into two categories: true cysts with epithelial lining and false/pseudocysts without epithelial lining, which is more common than true cysts [2]. Also, splenic cysts are divided into parasitic and non-parasitic. Nonparasitic cysts include primary with epithelial lining and secondary (pseudocyst) without epithelial lining (Figure 4) [2].

In fact, the differential diagnosis for a cystic splenic lesion is wide, and it requires consideration of various potential causes. It includes a congenital cyst, echinococcal disease, cystic lymphangioma/hemangioma, pyogenic splenic abscess, and cystic metastatic disease [5]. Given the wide range of potential causes, a thorough clinical assessment, imaging studies, and possibly further diagnostic procedures are essential for an accurate diagnosis. Collaboration between clinicians, radiologists, and pathologists is often crucial in determining the underlying cause of a cystic splenic lesion [4,6].

Due to a lack of specific signs and symptoms, around 30% to 45% of patients with splenic cysts are asymptomatic and present only due to abdominal mass. The clinical presentation correlates with the size of the lesion and its location [6]. Symptoms such as abdominal pain are more common in cysts larger than 6 cm, as in our case. Furthermore, some patients may exhibit respiratory symptoms such as pleuritic chest pain, persistent cough, and dyspnea [4]. Other uncommon symptoms, including nausea, vomiting, and dysphagia, are also described [2].

Splenic pseudocysts are more common than true cysts, representing up to 80% of the cases [7,8]. The most common cause for pseudocysts is resolved hematoma following blunt trauma. However, one-quarter of the cases may be due to infection, infarction, degenerative diseases, acute or chronic pancreatitis, and spontaneous subscapular hematoma [7,9]. Ultrasonography, contrast-enhanced computed tomography, and magnetic resonance imaging help determine the cyst characteristics, size, nature, morphology, and relation to surrounding tissue. A giant splenic pseudocyst refers to any pseudocyst with a diameter greater than 15 cm. However, histopathology is the ideal way to differentiate between the true cysts and the pseudocysts [2].

The primary objective in managing splenic pseudocysts is to alleviate symptoms while mitigating the risk of serious complications, such as overwhelming sepsis. Surgical intervention is typically considered when the pseudocysts exceed 5 cm in diameter or become symptomatic. The surgical approaches described in the literature include total splenectomy or more conservative interventions such as partial splenectomy, cyst aspiration, percutaneous drainage, partial cystectomy, fenestration, and marsupialization. Selecting the appropriate approach depends on various factors, including the cyst size, coverage by splenic parenchyma, residual splenic parenchyma, and relation to the hilum [10,11]. Although the concept of a spleen-preserving approach has become favorable over total splenectomy to avoid the risk of overwhelming life-threatening sepsis, total splenectomy is unavoidable for giant cysts, cysts with hilar location and/or cysts with splenic parenchymal coverage [2,3]. Partial splenectomy is recommended when a minimum of 25% of the spleen can be preserved. Compared to total and partial splenectomy, procedures such as cyst aspiration, percutaneous drainage, fenestration, and marsupialization are associated with a high rate of one-year recurrence [12,13].

Table 2 summarizes the published cases of non-traumatic splenic pseudocysts in the last 10 years.

**Table 2:** Summary of the clinical data of the published cases in the last 10 years. M, male; F, female; LUQ, left upper quadrant; TB, tuberculosis

Case	Author	Age (year)	Gender	Presenting complaint	Size (cm)	Management	Additional information
1	Soria-Céspede et al [1]	50	F	LUQ pain	9.5 × 9.0	Laparotomy and total Splenectomy	No history of trauma
2	Alhaddad et al. [2]	56	M	Left-sided back pain	9.8 × 9.5 × 9.0	Laparotomy with total splenectomy	No history of trauma
3	Pointer and Slakey [3]	34	F	Asymptomatic	7.8 × 8.8 × 9.6	Marsupialization of the cyst	No history of trauma
4	Sarwal et al. [14]	27	M	Asymptomatic	14 × 11	Laparoscopic splenectomy	No history of trauma
5	Kishanchand et al. [15]	30	F	LUQ pain and vomiting	10.6 × 9.6	Splenectomy	No history of trauma
6	Sefu Juma and Fauzia Ayubu [16]	35	F	LUQ pains	16 × 10	Total splenectomy	A cluster of daughter cysts is noticed
7	Cisse et al. [17]	20	F	Abdominal mass	20	Total splenectomy	No histories of trauma or infection
8	Yatham et al. [18]	38	M	LUQ pain and vomiting	16	Roux-en-Y splenocystojejunostomy	Resulted from erosion of the pancreatic tail pseudocyst into the splenic hilum
9	Alqahtani et al. [19]	26	M	Abdominal pain	15	Marsupialization and packing with the omentum	Following laparoscopic sleeve gastrectomy
10	Chakradhar et al. [20]	24	F	Upper abdominal pain, vomiting, and low-grade fever	16 × 10 × 8	Total splenectomy	Cytology findings were suggestive of TB of the spleen
11	Barajas-Puga et al. [21]	26	F	Asymptomatic	25 × 18 × 10	Open splenectomy	Splenic pseudocyst in a pregnant patient
12	Manoharan et al. [22]	50	M	Central abdominal pain shifted to the LUQ	10 × 9	Splenectomy	The spleen was situated in the false pelvis just above the bladder
13	Shrestha and Shrestha [23]	47	F	Intermittent LUQ pain	10 × 9 × 9	Splenectomy	-
14	Haddad et al. [24]	66	M	Early satiety, weight loss, and constipation	Not reported	Pancreatectomy and splenectomy	Splenic cyst communicating with a preexisting bilobed pancreatic tail cyst

Our patient presented with symptoms related to the giant size of the cyst, and his history was unremarkable to identify its etiology. He underwent cyst aspiration with partial cystectomy due to the location in the upper part and without parenchymal covering. On the 12-month follow-up, no recurrence was noted. This clinical report provides a preference for using a spleen-preserving approach as a way of managing splenic cysts since it reduces the risk of overwhelming sepsis and allows for a quicker recovery than total splenectomy. However, this case has some limitations, including its limited generalizability, the absence of a control group, and the lack of comprehensive investigations such as endoscopic ultrasonography. Furthermore, the patient's ongoing follow-up highlights the need for a more extended observation period to fully assess the long-term outcomes. Larger, controlled studies comparing different interventions will contribute to establishing the best practices.

## Conclusions

Despite being uncommon, splenic pseudocysts should be considered in the differential diagnosis of any splenic lesions. Furthermore, they should be distinguished from other benign/malignant splenic lesions as they require distinct management approaches. A definitive diagnosis is only achievable through histopathological investigation. We highly recommend the use of spleen-preserving interventions unless total splenectomy becomes unavoidable. Based on our experience and in the case of non-hilar, giant non-traumatic splenic pseudocysts without splenic parenchymal coverage, we suggest a cyst aspiration with cystectomy. Further studies are required to determine the superior intervention for non-traumatic splenic pseudocysts.
